# Diagnostic testing in people with primary ciliary dyskinesia: An international participatory study

**DOI:** 10.1371/journal.pgph.0001522

**Published:** 2023-09-11

**Authors:** Leonie Daria Schreck, Eva Sophie Lunde Pedersen, Isabelle Cizeau, Loretta Müller, Catherine Kruljac, Jane S. Lucas, Myrofora Goutaki, COVID-PCD patient advisory group, Claudia E. Kuehni

**Affiliations:** 1 Institute of Social and Preventive Medicine, University of Bern, Bern, Switzerland; 2 Graduate School for Health Sciences, University of Bern, Bern, Switzerland; 3 Association Dyskinésie Ciliaire Primitive, Saint-Étienne, France; 4 Department for BioMedical Research, University of Bern, Bern, Switzerland; 5 Division of Paediatric Respiratory Medicine and Allergology, Department of Paediatrics, University Hospital, Bern, Switzerland; 6 PCD Australia Primary Ciliary Dyskinesia, Altona Victoria, Australia; 7 Primary Ciliary Dyskinesia Centre, NIHR Biomedical Research Centre, University Hospital Southampton NHS Foundation Trust, Southampton, United Kingdom; 8 University of Southampton Faculty of Medicine, School of Clinical and Experimental Medicine, Southampton, United Kingdom; Purdue University, UNITED STATES

## Abstract

Diagnostic tests are important in primary ciliary dyskinesia (PCD), a rare disease, to confirm the diagnosis and characterize the disease. We compared diagnostic tests for PCD between countries worldwide, assessed whether people with PCD recall their tests, and identified factors associated with the use of tests. We used cross-sectional data from COVID-PCD—an international participatory cohort study collecting information directly from people with PCD. The baseline questionnaire inquired about tests used for PCD diagnosis. Using logistic regression, we investigated factors associated with measurement of nasal nitric oxide (nNO), biopsy for electron or video microscopy, and genetic testing. We included data from 747 participants (60% females) from 49 countries worldwide with median age 27 (interquartile range 12–44). Most (92%) reported diagnostic tests for PCD. Participants reported measurements of nNO (342; 49%), biopsy samples (561; 75%), and genetic tests (435; 58%). The reported use of individual tests, such as genetics, varied between countries from 38% in Switzerland to 68% in North America. Participant recall of test type also differed between countries with lowest recall in Switzerland. One-third (232; 36%) of participants reported all three tests (nNO, biopsy, and genetics). Recently diagnosed people reported more tests [nNO odds ratio (OR) 2.2, 95% Confidence Interval (CI) 1.5–3.2; biopsy OR 3.2, 95%CI 2.1–4.9; genetics OR 4.7, 95%CI 3.2–6.9] and those with situs abnormalities fewer tests (nNO OR 0.5, 95%CI 0.4–0.7; biopsy OR 0.5, 95%CI 0.4–0.8; genetics OR 0.7, 95%CI 0.5–0.94). Our results indicate PCD diagnostic testing differed widely around the world and many patients received incomplete diagnostic work-up based only on clinical features or single tests. People diagnosed long ago and those with situs abnormalities possibly benefit from supplementary testing to refine their diagnosis as a prerequisite for personalized medicine.

## Introduction

For people with primary ciliary dyskinesia (PCD), diagnostic tests are important to confirm the diagnosis and characterize the disease [1–3]. PCD is a rare heterogeneous genetic disease with an estimated prevalence of around 1:7,500 to 1:10,000 [4, 5]. Diagnosis relies on different tests, including measurement of nasal nitric oxide (nNO), assessment of ciliary function by high-speed video microscopy analysis (HSVA), visualisation of the ultrastructure by electron microscopy (EM) and immunofluorescence (IF), and genetic testing [6–8]. Multiple tests are usually needed to diagnose PCD; however, for a proportion of individuals, genetic testing or EM are sufficient as single tests to confirm PCD [9, 10]. Yet even among these people, a combination of different tests is important to understand associations between symptoms, defects in ciliary ultrastructure and function, and genotypes [1, 11]. Different disease phenotypes have different prognoses, they require adaptations in treatment plans and monitoring [6, 12]. Diagnostic characterization becomes increasingly important with regard to the development of personalized treatments [13] since only diagnostically well-characterized people with PCD will be eligible to participate in clinical trials and qualify for resulting treatments.

We know little about diagnostic testing for this rare disease in different countries. A survey among physicians from 194 paediatric PCD centres in 2007 looked at the availability of tests for PCD and showed that larger centres and those situated in countries with higher general government expenditures on health offered more tests [4, 14]. An international patient survey performed in 2014 [15] and a study from the international PCD (iPCD) cohort that analysed data collected until 2018 [16] also evaluated diagnostic testing among patients with PCD. Both studies focused on patients with PCD in Europe. The iPCD study investigated a previously recommended test combination, but not genetic tests use [16]. Diagnostic options for PCD have improved considerably over the past decade. Yet, we lack recent studies to understand what tests are actually performed, whether people with PCD know and remember tests, and what characteristics are associated with more comprehensive testing.

We analysed data from an ongoing international study of people with PCD to understand which diagnostic tests and test combinations were performed in various parts of the world, how well people with PCD recall their tests, and what factors explain using different tests.

## Materials and methods

### Study design and ethics

We used cross-sectional data from COVID-PCD—an ongoing participatory cohort study collecting information directly from people with PCD (clinicaltrials.gov registration number: NCT04602481). In collaboration with PCD patient support groups worldwide, the study was set up in spring 2020 to follow people with PCD throughout the COVID-19 pandemic and study other PCD-related research questions [17]. COVID-PCD questionnaires are available in five languages (English, French, German, Italian, Spanish) and completed anonymously online. In COVID-PCD, people with PCD or their caretakers, such as parents of children with PCD, actively contribute to study design and questionnaire content. Patient support groups advertised the study and motivated members to participate. We extracted data for analysis on October 20, 2022.

The Bern Cantonal Ethics Committee (Kantonale Ethikkomission Bern) in Switzerland (study ID: 2020–00830) approved the study. Participants provided informed consent when they registered for the study. We report according to Strengthening the Reporting of Observational Studies in Epidemiology (STROBE) recommendations [18].

### Study procedures

When participants register for the study, they complete a baseline questionnaire asking about country of residence, symptoms and clinical problems, such as situs abnormalities, and details about their PCD work-up, such as diagnostic tests, age, and year of PCD diagnosis. Participants enter data directly into a web-based database using the Research Electronic Data Capture (REDCap) platform [19], hosted by the Swiss medical registries and data linkage centre (SwissRDL) at the University of Bern. We included all COVID-PCD participants who completed the baseline questionnaire in our study and reported to have PCD.

### Information on diagnostic testing and participant characteristics

Baseline questionnaires asked participants about diagnostic testing and use of specific tests (nNO, biopsy, and genetic testing). If participants reported biopsy, we asked whether their sample was analysed via high-speed video microscopy (HSVA), electron microscopy (EM), or both. All questions were accompanied by short explanatory texts describing test procedures. We classified missing and “I don’t know” responses as “no recall”.

Participants also reported characteristics in the baseline questionnaire. We grouped the countries of residence Scotland, England, Wales, and Northern Ireland as the United Kingdom (UK). We combined the United States and Canada as North America since they use the same diagnostic algorithm and their PCD care is organized in a network [10]. We grouped countries with fewer than 25 participants together into either “other European countries” or “other non-European countries” (S1 Table). We categorized year of diagnosis into three periods (< = 2000, 2001–2010, > = 2011). We chose categories because of scarcity of diagnostic work-up before 2000, increased organization of PCD centres after 2010 [20], and the published consensus statement of the European Respiratory Society Task Force on PCD in children in 2009 [21]. We asked participants about organ situs. If they reported any organs in different positions, we classified this as situs abnormalities. We labelled missing values as “not reported”. We provide the questions in S2 Table.

### Statistical analyses

We described use and recall of diagnostic tests by country and region. For nNO, we only included participants aged > = 5 years—the age when this test is recommended and reliable [12]. We studied factors associated with reported tests (nNO, biopsy, and genetic testing) using multivariable logistic regression. Outcome was coded as 1 if participants reported that tests were performed; if participants answered “no”,”I don’t know”, or it was not reported, we coded as 0. Based on existing literature and expert knowledge, we included age at diagnosis, year of diagnosis, situs abnormalities, and country of residence as explanatory variables. For situs abnormalities, we combined the categories “no”, “I don’t know”, and “not reported” as “normal situs”. We performed a first sensitivity analysis excluding people who did not know or report their situs status and a second sensitivity analysis assuming the “no recall” group had the test done. To do so, we combined the “no recall” group with the group that indicated that the test was performed and compared them with people who reported no test. We used R version 4.2.0 for all analyses.

## Results

### Characteristics of the study population

We included 747 people of whom 446 (60%) were female. The median age at survey was 27 years [interquartile range (IQR) 12–44, range 0–85 years] (Table 1). Study participants came from 49 countries, most commonly from North America (158; 21%), the UK (150; 20%), and Germany (107; 14%). The median age at diagnosis was 8 years (IQR 2–19). About half (347; 46%) of participants were diagnosed after 2010, one-third (231; 31%) before 2001, 146 (20%) between 2001–2010, and 23 (3%) participants did not report year of diagnosis. About half (345; 46%) of participants reported situs abnormalities.

**Table 1:** Demographic information and clinical characteristics of the study population of people with primary ciliary dyskinesia (COVID-PCD study, N = 747).

		n (%)
**Sex**	Male	299 (40)
	Female	446 (60)
	Other	2 (0)^b^
**Age at survey**	Median (IQR, range)	27 (12–44, 0–85)
	0–19 y	270 (36)
	20–39 y	240 (32)
	40–59 y	192 (26)
	> 60 y	45 (6)
**Countries or regions** ^a^	North America	158 (21)
	United Kingdom	150 (20)
	Germany	107 (14)
	Italy	53 (7)
	Switzerland	48 (6)
	France	44 (6)
	Australia	33 (4)
	Other European countries	115 (15)
	Other non-European countries	39 (5)
**Age at diagnosis**	Median (IQR)	8 (2–19)
	Not reported	23 (3)
**Year of diagnosis**	Before 2001	231 (31)
	2001–2010	146 (20)
	After 2010	347 (46)
	Not reported	23 (3)
**Situs abnormalities**	Yes	345 (46)
	No	386 (52)
	I don’t know	15 (2)
	Not reported	1 (0)^b^
**Congenital heart defect**	Yes	58 (8)
	No	662 (89)
	I don’t know	23 (3)
	Not reported	4 (1)

Abbreviations: IQR, interquartile range. y, years. All characteristics are presented as n and column %, unless otherwise stated.

^a^Countries with N≥25 displayed in table, countries with N<25 were categorised into other European countries and other non-European countries.

^b^The percentages are greater than 0 but percentages < 0.5 have been rounded down to 0.

### Reported diagnostic testing

**Fig 1:**
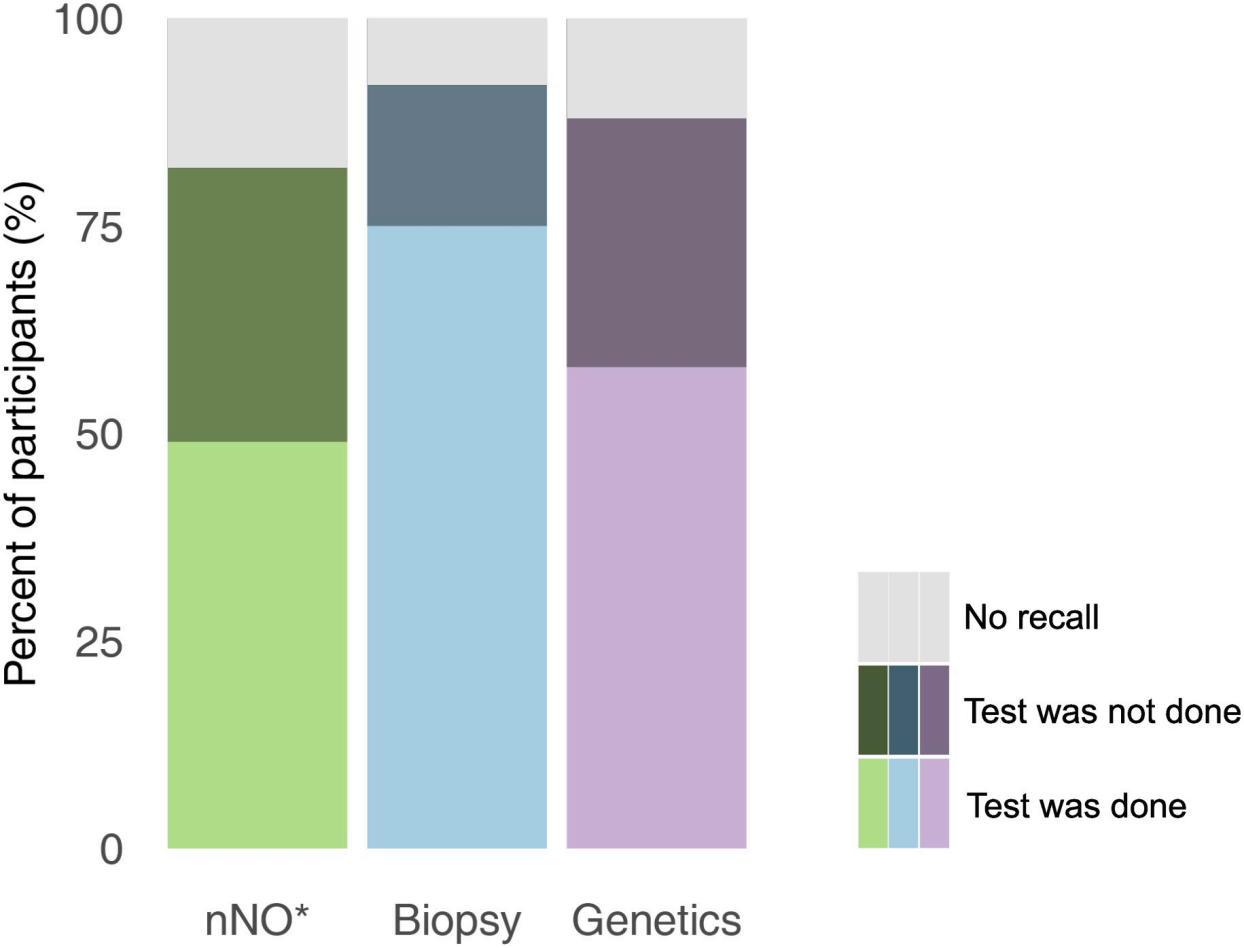
Diagnostic tests performed in people with primary ciliary dyskinesia (COVID-PCD study, N = 747). Abbreviations: nNO, nasal nitric oxide. *Only participants age > = 5 years are included (n = 693). Missing values and the answer category “I don’t know” were classified as “no recall” (missing values: nNO: n = 16, biopsy: n = 6, genetic testing: n = 9).

**Fig 2:**
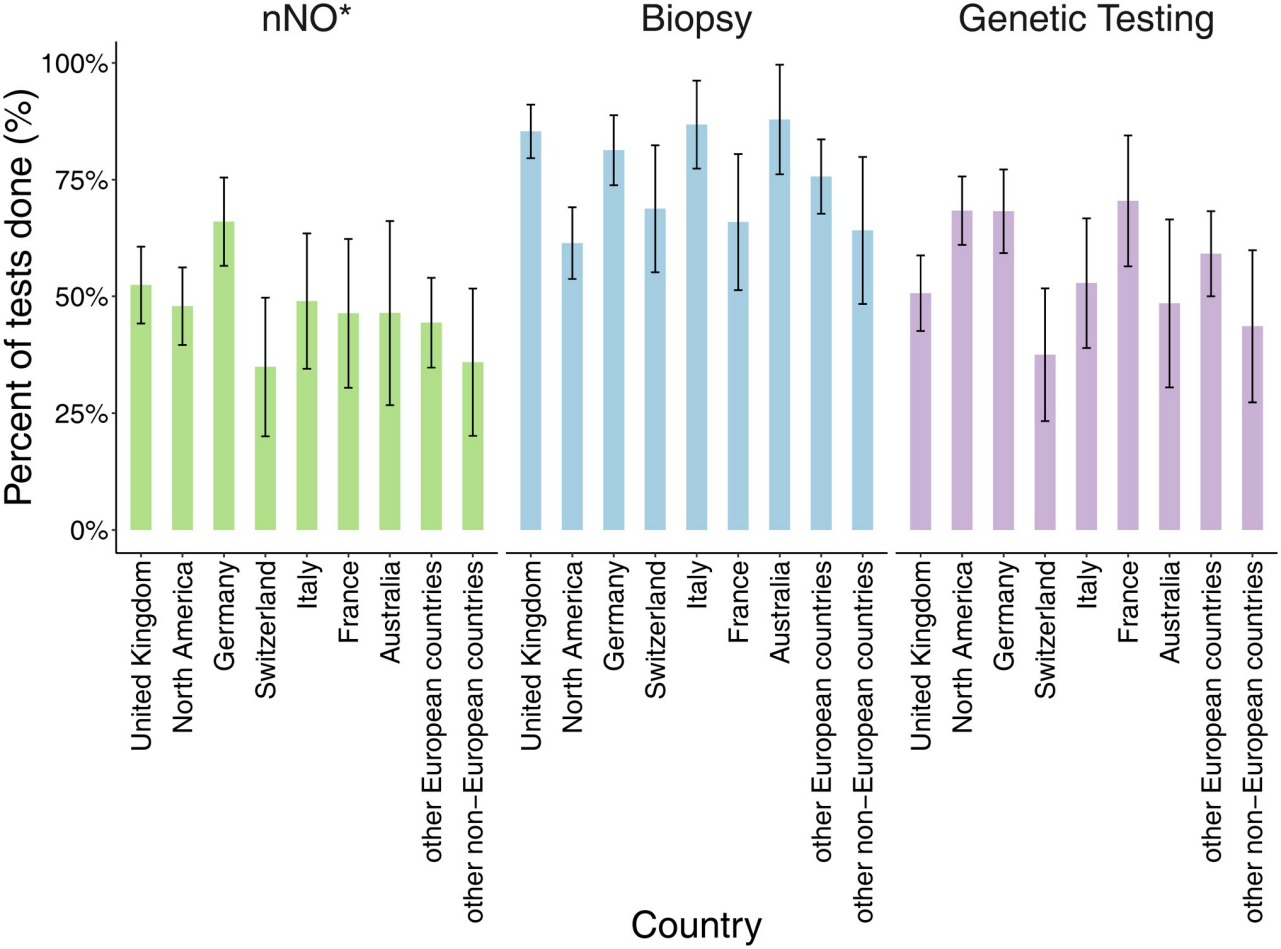
Diagnostic tests performed in people with primary ciliary dyskinesia, by country (COVID-PCD study, N = 747). Abbreviations: nNO, nasal nitric oxide. *Only participants age > = 5 years are included (n = 693).

Of the 747 participants, most (690; 92%) reported diagnostic testing for PCD (S3 Table). Nasal NO had been measured in 342 of 693 participants older than age 5 (49%). Participants reported biopsy samples (561; 75%) for electron or video microscopy, and genetic tests (435; 58%) (Fig 1). Among those reporting biopsy, 325 (58%) stated their sample was visualised with HSVA and 283 (50%) told it was further analysed using EM (S3 Table). Reported tests differed between countries (Fig 2). Biopsy was the most common test in all countries except for North America and France where genetic testing dominated. For participants older than age 5, nNO measurements were reported by 66 (66%) of 100 German participants, yet only by 15 (35%) of 43 people in Switzerland and 14 (36%) of 39 in other non-European countries. Biopsy samples were taken most often from people living in Australia (29; 88%), Italy (46; 87%), and the UK (128; 85%). Genetic testing was most often performed in France (31; 70%), Germany (73; 68%) and North America (108; 68%) and least often in Switzerland (18; 38%) and other non-European countries (17; 44%). Missing responses were fewer than 5% for all diagnostic tests.

### Recall of diagnostic testing

The proportion of people who did not remember whether a test was performed varied between tests (S3 Table); it was lowest for biopsy (57; 8%) and highest for nNO (125; 18%). Although people often knew that a biopsy sample was taken, they did not know whether it was analysed using HSVA (211; 38%) or EM (266; 47%).

The recall of tests also differed strongly between countries. Recall was poorest in Switzerland where 16 (37%) participants did not recall whether nNO was measured, 7 (15%) were unsure if biopsy samples were taken, and 10 (21%) did not know if genetic tests were performed.

### Combination of tests

**Fig 3:**
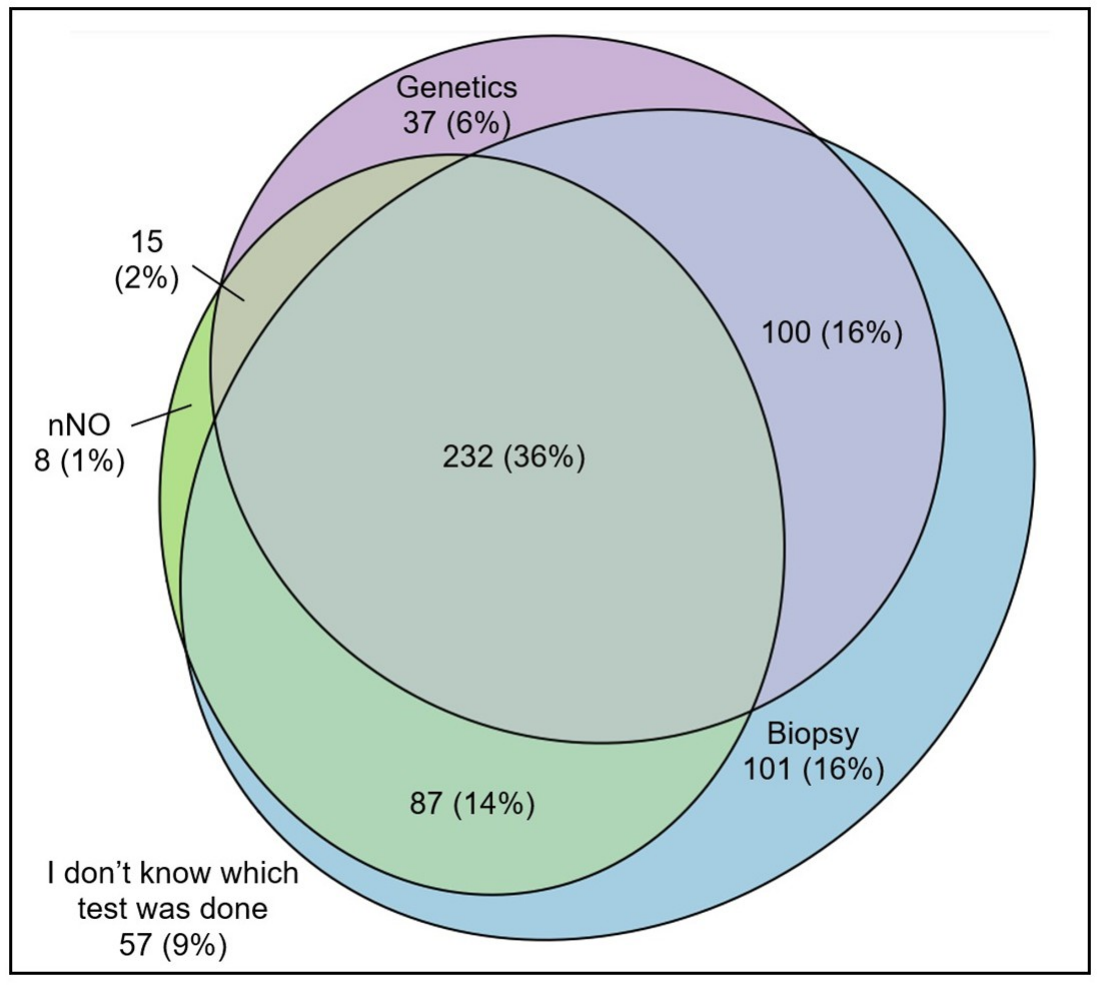
Venn diagram showing self-reported tests in people with primary ciliary dyskinesia who participated in COVID-PCD (*n* = 637) ^+^. Abbreviations: nNO, nasal nitric oxide. ^+^Participants under 5 years and/or who replied that no diagnostic was performed were excluded from this analysis.

Among participants older than age 5 with diagnostic tests, one-third (232; 36%) reported all three tests (nNO, biopsy, and genetics), 100 (16%) reported genetic testing and biopsy, and 101 (16%) reported biopsy alone (Fig 3). 57 (9%) did not remember which tests were performed. The frequency of combinations varied between countries. Most participants from Germany (53; 59%) reported combined analyses for nNO, biopsy, and genetic testing compared with only 7 (18%) in Switzerland and 6 (22%) in Australia (S3 Table). Of children younger than age 5, 39 (72%) reported the combination of biopsy and genetic testing.

### Predictors of diagnostic testing

**Fig 4:**
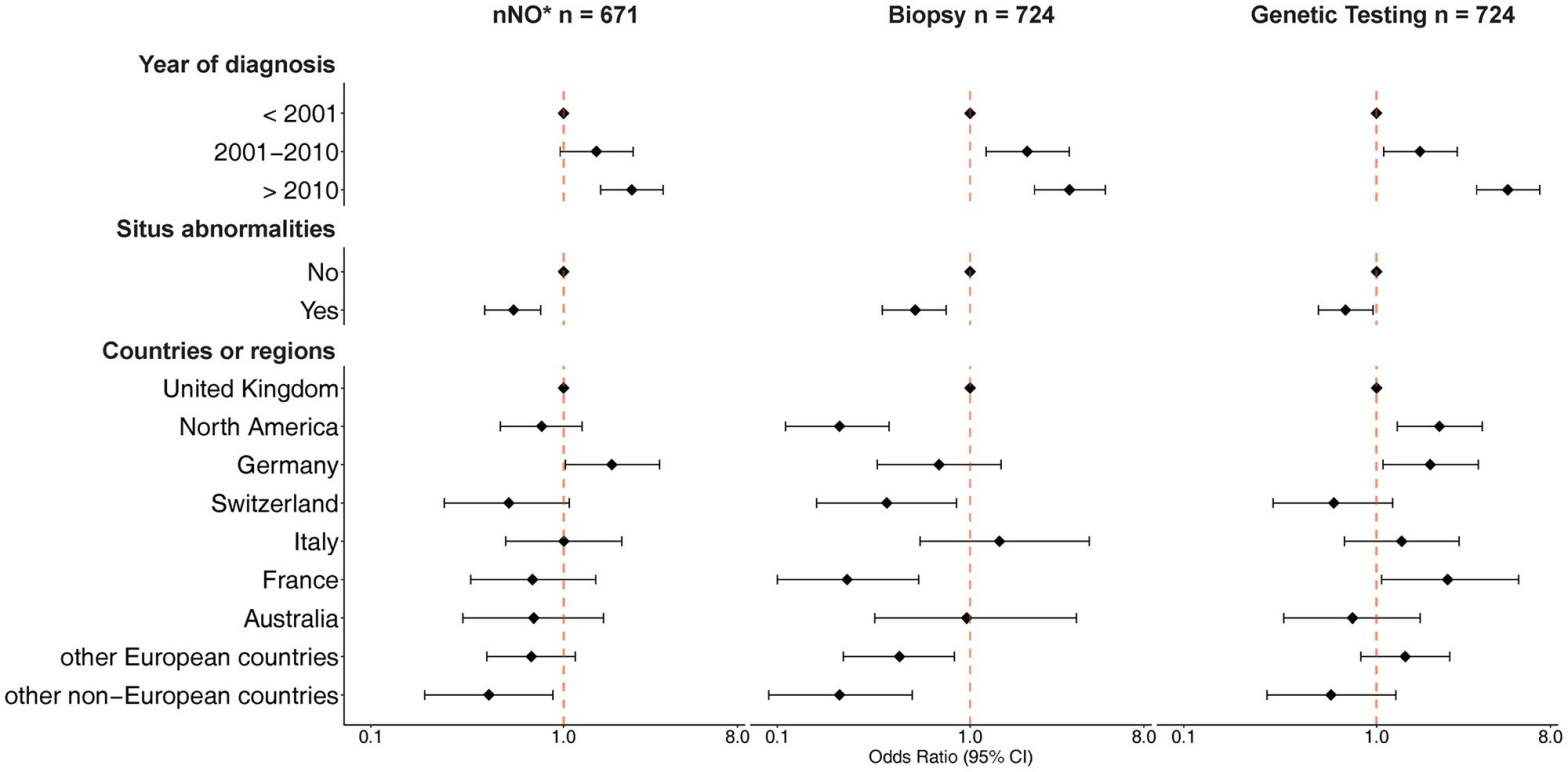
Factors associated with nNO measurement, biopsy and genetic tests in people with primary ciliary dyskinesia (COVID-PCD study). Abbreviations: CI, confidence interval. nNO, nasal nitric oxide. Participants who reported that the test was performed (“yes”) were compared to the group who reported either no test (“no”) or did not recall the test (“I don’t know” and missing). Odds Ratios are measures of associations between an exposure and an outcome. Odds ratios greater than 1 indicate that the outcome is more likely, odds ratios smaller than one that the outcome is less likely. Reading example: People who were diagnosed after 2010 have 2.2 higher odds of having nNO measured compared to people diagnosed before 2001. Odds Ratios were adjusted for all variables included in the model. *Only participants age > = 5 years are included.

Year of diagnosis and situs abnormalities were strongly associated with diagnostic testing (Fig 4). In a multivariable logistic regression, more people diagnosed after 2010 reported nNO measurement [Odds Ratio (OR) 2.2, 95% confidence interval (CI) 1.5–3.2], biopsy (OR 3.2, 95%CI 2.1–4.9), and genetic testing (OR 4.7, 95%CI 3.2–6.9) compared with people diagnosed before 2000. People with situs abnormalities reported fewer tests than people with situs solitus (nNO measurement: OR 0.5, 95%CI 0.4–0.7; biopsy: OR 0.5, 95%CI 0.4–0.8; genetic testing: OR 0.7, 95%CI 0.5–0.94). When compared with people from the UK, more Germans reported nNO measurements (OR 1.8, 95%CI 1.03–3.2), all countries (except for Italy) reported fewer biopsies, and participants from North America reported more genetic testing (OR 2.1, 95%CI 1.3–3.5). Age at diagnosis was not associated with any individual test; we excluded it from the final multivariable model (S7 Table). Results from the first sensitivity analysis, which excluded 16 people who did not know or report their situs status were similar to the main analysis (S8 Table). Our results also did not change in the second sensitivity analysis, which assumed that everybody in the group “no recall” had the test done (S5 Table).

## Discussion

### Summary of findings

Our international study of people with PCD found a large variation in diagnostic test use between countries. Even though most (92%) participants reported diagnostic testing for PCD, the use of individual tests varied widely. For example, only one-third of respondents from Switzerland reported genetic testing, whereas in North America two-thirds did. There were also differences in recall between countries. Overall, only one-third of participants older than age 5 reported all three tests (nNO measurement, biopsy, and genetics). People with PCD diagnosed after 2010 reported more tests and people with PCD who have situs abnormalities reported fewer tests.

### Strengths and limitations

With 747 enrolled participants, the COVID-PCD study is the largest study worldwide to collect data directly from people with PCD—it also enables comparisons between different regions of the world. The study includes the latest information about diagnostic testing among patients of all ages, including those who have not yet participated in clinical studies because they are treated by private physicians or reside in countries with decentralised PCD care. Another strength of the study is that it offers insight into what people with PCD know about their disease. However, self-reported data possibly leads to measurement bias, which is a limitation of our study. Since the study is anonymous, it was not possible to compare self-reported data with medical records. It might be participants were tested but not informed or forgot about tests. However, biopsies are uncomfortable, usually remembered procedures and genetic tests often need approval by patients and health insurance. When a nasal olive probe is inserted into one nostril during nNO measurement, it is also memorable. When we assumed tests were performed for all who reported no recall in a sensitivity analysis, the direction of the associations did not change, which strengthens the robustness of our findings. The immediate aim of the COVID-PCD study at development stage was to study how COVID-19 affected people with PCD. However, the study was set up with a broad scope to answer multiple research questions related to PCD. Therefore, the comprehensive baseline questionnaire was designed to allow collecting detailed information on diagnostic testing. The COVID-PCD study invites all people with PCD to participate. The study was advertised by patient support groups and was only available in five study languages. Participants who were not involved with support groups and those who do not speak the study languages could not be recruited. Since patients involved with support groups are usually better informed and treated than others who are not, our study may therefore have underestimated the general lack of diagnostic work-up. Due to the limited availability of study languages, our study is not representative of the target population in every country. However, the frequency of situs abnormalities and congenital heart defects (Table 1) in our study population was consistent with published data [22], suggesting participants were broadly representative in clinical terms.

### Comparison with other studies and interpretation

Genetic tests (58%) were more often reported in our study than in previous surveys. In a 2014 patient survey, only 39% reported genetic testing [15] and in the 2007 physician survey, 30% [4, 14]. Due to the cost-effectiveness of multigene panels, genetic tests have recently become widely available. Also, the number of known disease-causing genes has steadily increased, so genetic testing can currently identify genes for more than 70% of people with PCD [12, 23]. The large variation between countries reflects historical conditions or country-specific opportunities. In North America, genetic testing has been a cornerstone of the diagnostic algorithm for years, while in many European countries, PCD diagnosis has been based primarily on EM and HSVA. Projects such as the 100,000 Genomes Project in the UK led to a local increase of genetic tests [23–25]. In other countries, availability of genetic testing is still limited for PCD diagnostics and rare diseases in general [26, 27]. In many countries, structural changes in healthcare systems would be needed to improve access to genetic testing, which is a slow process that unfortunately limits care and equal opportunities for patients with rare diseases worldwide [28].

Recall of diagnostic tests varied between countries. It was overall lowest in Switzerland—the country with the second highest health expenditure per capita in the world since 1990, after the United States [29]. PCD care in Switzerland is decentralised and many patients with PCD are treated by physicians who are not PCD specialists. Therefore, patients have insufficient information and education. For people with rare diseases, patient empowerment and shared decision-making are essential. For shared decision-making, patients must become experts as many encounter health care professionals with little expertise on their disease, particularly in emergency situations [30]. Our findings indicate respondents’ knowledge about their disease is limited, which highlights the need for better information, education, and empowerment.

Although we showed test use differed between countries, diagnostic testing also depended strongly on year of diagnosis and situs. Age at diagnosis was not associated with the use of tests, which suggests diagnostic work-up is not more complete in paediatric compared with adult settings—a finding that contrasts better expected PCD awareness among paediatric pulmonologists. Our results suggest only newly diagnosed people with PCD benefitted from more comprehensive testing and newer tests, such as genetics. Although people diagnosed before 2000 often only received partial diagnostic work-up, they were not later recalled for supplementary testing to confirm and refine diagnoses. The same is true for people with PCD with situs abnormalities—physicians may be satisfied with clinical diagnosis and consider PCD proven. Thus, they do not offer biopsy or genetic testing to confirm diagnosis, which is insufficient because only 20–25% of people with situs inversus have PCD [31].

## Conclusion

We found PCD diagnostics differed markedly around the world and many people with PCD still have incomplete diagnostic work-up with diagnoses based only on clinical features or single tests. Several pre-clinical studies are developing novel molecular therapies for PCD; thus, understanding individual genotypes will become imperative for treatment. Therefore, it is important clinicians review tests and results with patients and plan further tests, if necessary. People diagnosed with PCD long ago and those with situs abnormalities possibly benefit from supplementary testing to improve diagnostic characterization as a prerequisite for personalized medicine.

## Supporting information

S1 Table Countries of residence of COVID-PCD participants with less than 25 participants who were grouped into other European countries and other non-European countries. (DOCX)

S2 Table Formulation of questions and answers from the English adult baseline questionnaire of the COVID-PCD study. (DOCX)

S3 Table Diagnostic tests performed in people with primary ciliary dyskinesia (PCD), by country^a^ (COVID-PCD study, N = 747). Abbreviations: nNO, nasal nitric oxide. CI, confidence interval. HSVA, high-speed video microscopy. EM, electron microscopy. Missing values and the answer category “I don’t know” were classified as “no recall” (missing values: nNO: n = 16, biopsy: n = 6, genetic testing: n = 9, HSVA: n = 1, EM: n = 1). All characteristics are presented as n and column %, unless otherwise stated. ^a^Countries with N≥25 displayed in table, countries with N<25 were categorised into other European countries and other non-European countries. ^b^Only participants age > = 5 years are included. ^c^Proportions of HSVA and EM are calculated out of people who report a biopsy. ^d^Tests combination refers to nNO, biopsy and genetics performed, only participants age > = 5 years who report any diagnostic test done were included (n = 637).
(DOCX)

S4 Table Factors associated with nNO measurement, biopsy and genetic tests, in people with primary ciliary dyskinesia (PCD) (COVID-PCD study). Abbreviations: nNO, nasal nitric oxide. Odds Ratio (OR) and 95% Confidence Interval (CI) reported. Odds Ratios were adjusted for all variables included in the multivariable model. Performed tests: Participants who report that the test was performed (“yes”) were compared to the group who reported either no test (“no”) or did not recall the test (“I don’t know” and missing). ^a^Only participants age > = 5 years are included.
(DOCX)

S5 Table Sensitivity analysis of factors associated with performed nNO, biopsy and genetic testing in which we assumed that everybody in the group “no recall” had the test done in the COVID-PCD study^a^. Abbreviations: nNO, nasal nitric oxide. Adjusted Odds Ratio (OR) and 95% Confidence Interval (CI) reported. Odds Ratios were adjusted for all variables included in the multivariable model. ^a^Participants who report that the test was performed (“yes”) and who did not recall the test (“I don’t know” and missing) were compared to the group who reported no test (“no”). ^b^Only participants age > = 5 years are included.
(DOCX)

S6 Table Performance of nNO measurement, biopsy and genetic tests by year of diagnosis, situs abnormalities and countries, in people with primary ciliary dyskinesia (PCD) (COVID-PCD study). Abbreviations: nNO, nasal nitric oxide. Performed tests: Participants who report that the test was performed (“yes”) were compared to the group who reported either no test (“no”) or did not recall the test (“I don’t know” and missing). ^a^Only participants age > = 5 years are included.
(DOCX)

S7 Table Factors associated with nNO measurement, biopsy and genetic tests, in people with primary ciliary dyskinesia (PCD), univariable (COVID-PCD study, N = 747)^a^. Abbreviations: nNO, nasal nitric oxide. Odds Ratio (OR) and 95% Confidence Interval (CI) reported. Performed tests: Participants who report that the test was performed (“yes”) were compared to the group who reported either no test (“no”) or did not recall the test (“I don’t know” and missing). ^a^Univariable analysis. ^b^Only participants age > = 5 years are included.
(DOCX)

S8 Table Sensitivity analysis of factors associated with nNO measurement, biopsy, and genetic tests, in people with primary ciliary dyskinesia (PCD) who answered “yes” or “no” to the question about organ situs (COVID-PCD study). Abbreviations: nNO, nasal nitric oxide. Odds Ratio (OR) and 95% Confidence Interval (CI) reported. Odds Ratios were adjusted for all variables included in the multivariable model. Performed tests: Participants who report that the test was performed (“yes”) were compared to the group who reported either no test (“no”) or did not recall the test (“I don’t know” and missing). aOnly participants age > = 5 years are included.
(DOCX)

## References

[1] Goutaki M, Pedersen ESL. Phenotype-genotype associations in primary ciliary dyskinesia: where do we stand? The European respiratory journal. 2021;58(2). doi: 10.1183/13993003.00392-2021 34353866

[2] Brennan SK, Ferkol TW, Davis SD. Emerging Genotype-Phenotype Relationships in Primary Ciliary Dyskinesia. International journal of molecular sciences. 2021;22(15). doi: 10.3390/ijms22158272 34361034 PMC8348038

[3] Davis SD, Ferkol TW, Rosenfeld M, Lee HS, Dell SD, Sagel SD, et al. Clinical features of childhood primary ciliary dyskinesia by genotype and ultrastructural phenotype. American journal of respiratory and critical care medicine. 2015;191(3):316–24. doi: 10.1164/rccm.201409-1672OC 25493340 PMC4351577

[4] Kuehni CE, Frischer T, Strippoli MP, Maurer E, Bush A, Nielsen KG, et al. Factors influencing age at diagnosis of primary ciliary dyskinesia in European children. The European respiratory journal. 2010;36(6):1248–58. doi: 10.1183/09031936.00001010 20530032

[5] Hannah WB, Seifert BA, Truty R, Zariwala MA, Ameel K, Zhao Y, et al. The global prevalence and ethnic heterogeneity of primary ciliary dyskinesia gene variants: a genetic database analysis. The Lancet Respiratory medicine. 2022. doi: 10.1016/S2213-2600(21)00453-7 35051411 PMC9064931

[6] Goutaki M, Shoemark A. Diagnosis of Primary Ciliary Dyskinesia. Clinics in chest medicine. 2022;43(1):127–40. doi: 10.1016/j.ccm.2021.11.008 35236553

[7] Jackson CL, Behan L, Collins SA, Goggin PM, Adam EC, Coles JL, et al. Accuracy of diagnostic testing in primary ciliary dyskinesia. The European respiratory journal. 2016;47(3):837–48. doi: 10.1183/13993003.00749-2015 26647444 PMC4771621

[8] Kuehni CE, Lucas JS. Toward an Earlier Diagnosis of Primary Ciliary Dyskinesia. Which Patients Should Undergo Detailed Diagnostic Testing? Ann Am Thorac Soc. 2016;13(8):1239–43. doi: 10.1513/AnnalsATS.201605-331PS 27258773

[9] Lucas JS, Barbato A, Collins SA, Goutaki M, Behan L, Caudri D, et al. European Respiratory Society guidelines for the diagnosis of primary ciliary dyskinesia. The European respiratory journal. 2017;49(1). 10.1183/13993003.01090-2016 PMC6054534 27836958

[10] Shapiro AJ, Davis SD, Polineni D, Manion M, Rosenfeld M, Dell SD, et al. Diagnosis of Primary Ciliary Dyskinesia. An Official American Thoracic Society Clinical Practice Guideline. American journal of respiratory and critical care medicine. 2018;197(12):e24–e39. doi: 10.1164/rccm.201805-0819ST 29905515 PMC6006411

[11] Shoemark A, Rubbo B, Legendre M, Fassad MR, Haarman EG, Best S, et al. Topological data analysis reveals genotype-phenotype relationships in primary ciliary dyskinesia. The European respiratory journal. 2021;58(2). doi: 10.1183/13993003.02359-2020 33479112

[12] Lucas JS, Davis SD, Omran H, Shoemark A. Primary ciliary dyskinesia in the genomics age. The Lancet Respiratory medicine. 2020;8(2):202–16. doi: 10.1016/S2213-2600(19)30374-1 31624012

[13] Paff T, Omran H, Nielsen KG, Haarman EG. Current and Future Treatments in Primary Ciliary Dyskinesia. International journal of molecular sciences. 2021;22(18). doi: 10.3390/ijms22189834 34575997 PMC8470068

[14] Strippoli MP, Frischer T, Barbato A, Snijders D, Maurer E, Lucas JS, et al. Management of primary ciliary dyskinesia in European children: recommendations and clinical practice. The European respiratory journal. 2012;39(6):1482–91. doi: 10.1183/09031936.00073911 22282549

[15] Behan L, Dunn Galvin A, Rubbo B, Masefield S, Copeland F, Manion M, et al. Diagnosing primary ciliary dyskinesia: an international patient perspective. The European respiratory journal. 2016;48(4):1096–107. doi: 10.1183/13993003.02018-2015 27492837 PMC5045441

[16] Halbeisen FS, Shoemark A, Barbato A, Boon M, Carr S, Crowley S, et al. Time trends in diagnostic testing for primary ciliary dyskinesia in Europe. The European respiratory journal. 2019;54(4). doi: 10.1183/13993003.00528-2019 31273043

[17] Pedersen ESL, Collaud ENR, Mozun R, Ardura-Garcia C, Lam YT, Harris A, et al. COVID-PCD: a participatory research study on the impact of COVID-19 in people with primary ciliary dyskinesia. ERJ open research. 2021;7(1). 10.1183/23120541.00843-2020 PMC7983255 33778058

[18] von Elm E, Altman DG, Egger M, Pocock SJ, Gøtzsche PC, Vandenbroucke JP. The Strengthening the Reporting of Observational Studies in Epidemiology (STROBE) statement: guidelines for reporting observational studies. J Clin Epidemiol. 2008;61(4):344–9. doi: 10.1016/j.jclinepi.2007.11.008 18313558

[19] Harris PA, Taylor R, Thielke R, Payne J, Gonzalez N, Conde JG. Research electronic data capture (REDCap)—a metadata-driven methodology and workflow process for providing translational research informatics support. Journal of biomedical informatics. 2009;42(2):377–81. doi: 10.1016/j.jbi.2008.08.010 18929686 PMC2700030

[20] Lucas JS, Chetcuti P, Copeland F, Hogg C, Kenny T, Moya E, et al. Overcoming challenges in the management of primary ciliary dyskinesia: the UK model. Paediatric respiratory reviews. 2014;15(2):142–5. doi: 10.1016/j.prrv.2013.04.007 23764568

[21] Barbato A, Frischer T, Kuehni CE, Snijders D, Azevedo I, Baktai G, et al. Primary ciliary dyskinesia: a consensus statement on diagnostic and treatment approaches in children. The European respiratory journal. 2009;34(6):1264–76. doi: 10.1183/09031936.00176608 19948909

[22] Goutaki M, Meier AB, Halbeisen FS, Lucas JS, Dell SD, Maurer E, et al. Clinical manifestations in primary ciliary dyskinesia: systematic review and meta-analysis. The European respiratory journal. 2016;48(4):1081–95. doi: 10.1183/13993003.00736-2016 27492829

[23] Wheway G, Thomas NS, Carroll M, Coles J, Doherty R, Genomics England Research C, et al. Whole genome sequencing in the diagnosis of primary ciliary dyskinesia. BMC Med Genomics. 2021;14(1):234. doi: 10.1186/s12920-021-01084-w 34556108 PMC8461892

[24] Smedley D, Smith KR, Martin A, Thomas EA, McDonagh EM, Cipriani V, et al. 100,000 Genomes Pilot on Rare-Disease Diagnosis in Health Care—Preliminary Report. The New England journal of medicine. 2021;385(20):1868–80. doi: 10.1056/NEJMoa2035790 34758253 PMC7613219

[25] Shoemark A, Griffin H, Wheway G, Hogg C, Lucas JS, Camps C, et al. Genome sequencing reveals underdiagnosis of primary ciliary dyskinesia in bronchiectasis. The European respiratory journal. 2022. doi: 10.1183/13993003.00176-2022 35728977

[26] Álvaro-Sánchez S, Abreu-Rodríguez I, Abulí A, Serra-Juhe C, Garrido-Navas MDC. Current Status of Genetic Counselling for Rare Diseases in Spain. Diagnostics (Basel, Switzerland). 2021;11(12). doi: 10.3390/diagnostics11122320 34943558 PMC8700506

[27] Robillard JM, Feng TL, Kabacińska K. Access to genetic testing for rare diseases: Existing gaps in public-facing information. World medical & health policy. 2021;13(3):518–25. doi: 10.1002/wmh3.469 34692184 PMC8518969

[28] Schee Genannt Halfmann S, Mählmann L, Leyens L, Reumann M, Brand A. Personalized Medicine: What’s in it for Rare Diseases? Advances in experimental medicine and biology. 2017;1031:387–404. doi: 10.1007/978-3-319-67144-4_22 29214584

[29] World Health Organization. Global Health Expenditure Database [cited 20 December 2022]. https://apps.who.int/nha/database/Country_Profile/Index/en.

[30] De Santis M, Hervas C, Weinman A, Bosi G, Bottarelli V. Patient empowerment of people living with rare diseases. Its contribution to sustainable and resilient healthcare systems. Annali dell’Istituto superiore di sanita. 2019;55(3):283–91. doi: 10.4415/ANN_19_03_15 31553324

[31] Aylsworth AS. Clinical aspects of defects in the determination of laterality. Am J Med Genet. 2001;101(4):345–55. 11471158

