# Supplementary material for: Diagnostic testing in people with primary ciliary dyskinesia: An international participatory study
Source: PLOS Glob Public Health. 2023 Sep 11;3(9):e0001522. doi: 10.1371/journal.pgph.0001522 (PMC10495017; doi:10.1371/journal.pgph.0001522)
Supplement: S1 Table — (DOCX) [file pgph.0001522.s001.docx]

**S1 Table.** Countries of residence of COVID-PCD participants with less than 25 participants who were grouped into other European countries and other non-European countries

| **Country** | **Number of participants** |
| --- | --- |
| **Other European countries** | **115** |
| Austria | 7 |
| Belgium | 9 |
| Croatia | 1 |
| Cyprus | 5 |
| Czech Republic | 1 |
| Denmark | 10 |
| Finland | 1 |
| Georgia | 2 |
| Greece | 1 |
| Hungary | 1 |
| Ireland | 9 |
| Jersey | 1 |
| Netherlands | 21 |
| Norway | 12 |
| Poland | 5 |
| Portugal | 3 |
| Romania | 1 |
| Spain | 16 |
| Sweden | 9 |
|  |  |
| **Other non-European countries** | **39** |
| Argentina | 1 |
| Bahrain | 2 |
| Brazil | 3 |
| Cameroon | 1 |
| Chile | 1 |
| Colombia | 1 |
| Ecuador | 1 |
| Hong Kong | 1 |
| India | 2 |
| Iran | 1 |
| Israel | 6 |
| Kuwait | 2 |
| Lebanon | 1 |
| Mexico | 2 |
| New Zealand | 1 |
| Panama | 1 |
| Puerto Rico | 2 |
| South Africa | 6 |
| Turkey | 3 |
| other country | 1 |
