# Supplementary material for: Diagnostic testing in people with primary ciliary dyskinesia: An international participatory study
Source: PLOS Glob Public Health. 2023 Sep 11;3(9):e0001522. doi: 10.1371/journal.pgph.0001522 (PMC10495017; doi:10.1371/journal.pgph.0001522)
Supplement: S2 Table — (DOCX) [file pgph.0001522.s002.docx]

**S2 Table.** Formulation of questions and answers from the English adult baseline questionnaire of the COVID-PCD study

| **Question** | **Answer category** |
| --- | --- |
| *Countries and regions* | |
| Which country do you live in? | List of all countries worldwide |
| Which other country? | text |
| *Situs inversus* | |
| Are any of your organs in a different position compared to most people? (E.g. the heart on the right side instead of on the left) | No  Yes  I don’t know |
| *Diagnostic testing* | |
| Have you had diagnostic tests for PCD? | No Yes |
| Have you had a nasal nitric oxide test? (This test measures a gas from the nose through a thin tube that leads to a computer) | No Yes I don't know/I cannot remember |
| What was the nasal nitric oxide test result? | Normal Suggestive of PCD (very low) Borderline/unclear I don't know/I cannot remember |
| Have you had a nasal brush biopsy? (Uncomfortable scraping or brushing to collect cilia/hair cells from the nose, or a brushing to collect cells from the airways during a bronchoscopy) | No Yes I don't know/I cannot remember |
| Do you know if the sample was tested by high speed video microscopy? (The sample was looked at under a microscope to see how the cilia/hairs move) | No, it was not tested by high speed video microscopy Yes, it was tested by high speed video microscopy I don't know/ I cannot remember |
| What was the result of the high speed video microscopy? | Static, slow, or abnormal movement, typical for PCD Normal movement Unclear result I don't know/ I cannot remember |
| Do you know if the sample was tested by electron microscopy? (The sample was looked at with a microscope to see the inside structure of the cilia/hairs) | No, it was not tested by electron microscopy Yes, it was tested by electron microscopy I don't know/ I cannot remember |
| What was the result of the electron microscopy? | Typical for PCD Normal Unclear result I don't know/I cannot remember |
| Have you had a genetic test (looking for genes that cause PCD)? | No Yes I don't know/ I cannot remember |
| Were any genes found that cause PCD? | No Yes I don't know/ I cannot remember/ waiting for results |
| *Year of diagnosis* | |
| Which year were you diagnosed with PCD? | text (integer, Min: 1900, Max: 2025) |
| How old were you, when you were diagnosed with PCD? | text (integer, Min: 0, Max: 110) |
