# Supplementary material for: Diagnostic testing in people with primary ciliary dyskinesia: An international participatory study
Source: PLOS Glob Public Health. 2023 Sep 11;3(9):e0001522. doi: 10.1371/journal.pgph.0001522 (PMC10495017; doi:10.1371/journal.pgph.0001522)
Supplement: S3 Table — Abbreviations: nNO, nasal nitric oxide. CI, confidence interval. HSVA, high-speed video microscopy. EM, electron microscopy. Missing values and the answer category “I don’t know” were classified as “no recall” (missing values: nNO: n = 16, biopsy: n = 6, genetic testing: n = 9, HSVA: n = 1, EM: n = 1). All characteristics are presented as n and column %, unless otherwise stated. aCountries with N≥25 displayed in table, countries with N<25 were categorised into other European countries and other non-European countries. bOnly participants age > = 5 years are included. cProportions of HSVA and EM are calculated out of people who report a biopsy. dTests combination refers to nNO, biopsy and genetics performed, only participants age > = 5 years who report any diagnostic test done were included (n = 637). (DOCX) [file pgph.0001522.s003.docx]

**S3 Table.** Diagnostic tests performed in people with primary ciliary dyskinesia (PCD), by country^a^ (COVID-PCD study, N = 747)

|  | **Total** | **United Kingdom** | **North America** | **Germany** | **Italy** | **Switzerland** | **France** | **Australia** | **other European countries**^+^ | **other non-European countries**^+^ |
| --- | --- | --- | --- | --- | --- | --- | --- | --- | --- | --- |
|  | N = 747 | *n* = 150 | *n* = 158 | *n* = 107 | *n* = 53 | *n* = 48 | *n* = 44 | *n* = 33 | *n* = 115 | *n* = 39 |
|  | n (%) | n (%) | n (%) | n (%) | n (%) | n (%) | n (%) | n (%) | n (%) | n (%) |
| **Any diagnostic test** |  |  |  |  |  |  |  |  |  |  |
| Done | 690 (92) | 144 (96) | 145 (92) | 97 (91) | 49 (92) | 43 (90) | 39 (89) | 32 (97) | 109 (95) | 32 (82) |
| Not done | 51 (7) | 4 (3) | 12 (8) | 8 (7) | 4 (8) | 4 (8) | 5 (11) | 1 (3) | 6 (5) | 7 (18) |
| No recall | 6 (1) | 2 (1) | 1 (1) | 2 (2) | 0 (0) | 1 (2) | 0 (0) | 0 (0) | 0 (0) | 0 (0) |
|  |  |  |  |  |  |  |  |  |  |  |
| **nNO^b^** | n = 693 | n = 145 | n = 142 | n = 100 | n = 49 | n = 43 | n = 41 | n = 28 | n = 106 | n = 39 |
| Done [n (%, 95% CI)] | 342 (49) | 76 (52, 44-61) | 68 (48, 40-56) | 66 (66, 57-75) | 24 (49, 34-63) | 15 (35, 20-50) | 19 (46, 30-62) | 13 (46, 26-66) | 47 (44, 35-54) | 14 (36, 20-52) |
| Not done | 226 (33) | 39 (27) | 49 (35) | 22 (22) | 19 (39) | 12 (28) | 17 (41) | 11 (39) | 38 (36) | 19 (49) |
| No recall | 125 (18) | 30 (21) | 25 (18) | 12 (12) | 6 (12) | 16 (37) | 5 (12) | 4 (14) | 21 (20) | 6 (15) |
|  |  |  |  |  |  |  |  |  |  |  |
| **Biopsy** |  |  |  |  |  |  |  |  |  |  |
| Done [n (%, 95% CI)] | 561 (75) | 128 (85, 80-91) | 97 (61, 54-69) | 87 (81, 74-89) | 46 (87, 77-96) | 33 (69, 55-82) | 29 (66, 51-81) | 29 (88, 76-100) | 87 (76, 68-84) | 25 (64, 48-80) |
| Not done | 129 (17) | 9 (6) | 47 (30) | 17 (16) | 6 (11) | 8 (17) | 9 (20) | 3 (9) | 19 (17) | 11 (28) |
| No recall | 57 (8) | 13 (9) | 14 (9) | 3 (3) | 1 (2) | 7 (15) | 6 (14) | 1 (3) | 9 (8) | 3 (8) |
|  |  |  |  |  |  |  |  |  |  |  |
| **Genetics** |  |  |  |  |  |  |  |  |  |  |
| Done [n (%, 95% CI)] | 435 (58) | 76 (51, 43-59) | 108 (68, 61-76) | 73 (68, 59-77) | 28 (53, 38-66) | 18 (38, 23-52) | 31 (70, 57-85) | 16 (48, 30-66) | 68 (59, 50-68) | 17 (44, 27-60) |
| Not done | 223 (30) | 45 (30) | 37 (23) | 28 (26) | 16 (30) | 20 (42) | 11 (25) | 12 (36) | 34 (30) | 20 (51) |
| No recall | 89 (12) | 29 (19) | 13 (8) | 6 (6) | 9 (17) | 10 (21) | 2 (5) | 5 (15) | 13 (11) | 2 (5) |
|  |  |  |  |  |  |  |  |  |  |  |
| **HSVA^c^** | n = 561 | n = 128 | n = 97 | n = 87 | n = 46 | n = 33 | n = 29 | n = 29 | n = 87 | n = 25 |
| Done | 325 (58) | 61 (48) | 40 (41) | 73 (84) | 30 (65) | 22 (67) | 20 (69) | 21 (72) | 43 (49) | 15 (60) |
| Not done | 25 (4) | 3 (2) | 9 (9) | 2 (2) | 0 (0) | 0 (0) | 0 (0) | 1 (3) | 7 (8) | 3 (12) |
| No recall | 211 (38) | 64 (50) | 48 (49) | 12 (14) | 16 (35) | 11 (33) | 9 (31) | 7 (24) | 37 (43) | 7 (28) |
|  |  |  |  |  |  |  |  |  |  |  |
| **EM^c^** | n = 561 | n = 128 | n = 97 | n = 87 | n = 46 | n = 33 | n = 29 | n = 29 | n = 87 | n = 25 |
| Done | 283 (50) | 59 (46) | 46 (47) | 56 (64) | 26 (57) | 16 (48) | 14 (48) | 16 (55) | 38 (44) | 12 (48) |
| Not done | 12 (2) | 2 (2) | 1 (1) | 5 (6) | 0 (0) | 0 (0) | 0 (0) | 0 (0) | 1 (1) | 3 (12) |
| No recall | 266 (47) | 67 (52) | 50 (52) | 26 (30) | 20 (43) | 17 (52) | 15 (52) | 13 (45) | 48 (55) | 10 (40) |
|  |  |  |  |  |  |  |  |  |  |  |
| **test com-bination done^d^** | 232 (36) | 48 (35) | 49 (38) | 53 (59) | 14 (31) | 7 (18) | 16 (44) | 6 (22) | 30 (30) | 9 (28) |

Abbreviations: nNO, nasal nitric oxide. CI, confidence interval. HSVA, high-speed video microscopy. EM, electron microscopy. Missing values and the answer category “I don’t know” were classified as “no recall” (missing values: nNO: n = 16, biopsy: n = 6, genetic testing: n = 9, HSVA: n = 1, EM: n = 1). All characteristics are presented as n and column %, unless otherwise stated. ^a^Countries with N≥25 displayed in table, countries with N<25 were categorised into other European countries and other non-European countries. ^b^Only participants age >= 5 years are included. ^c^Proportions of HSVA and EM are calculated out of people who report a biopsy. ^d^Tests combination refers to nNO, biopsy and genetics performed, only participants age >=5 years who report any diagnostic test done were included (n = 637).
