# Supplementary material for: Diagnostic testing in people with primary ciliary dyskinesia: An international participatory study
Source: PLOS Glob Public Health. 2023 Sep 11;3(9):e0001522. doi: 10.1371/journal.pgph.0001522 (PMC10495017; doi:10.1371/journal.pgph.0001522)
Supplement: S4 Table — Abbreviations: nNO, nasal nitric oxide. Odds Ratio (OR) and 95% Confidence Interval (CI) reported. Odds Ratios were adjusted for all variables included in the multivariable model. Performed tests: Participants who report that the test was performed (“yes”) were compared to the group who reported either no test (“no”) or did not recall the test (“I don’t know” and missing). aOnly participants age > = 5 years are included. (DOCX) [file pgph.0001522.s004.docx]

**S4 Table.** Factors associated with nNO measurement, biopsy and genetic tests, in people with primary ciliary dyskinesia (PCD) (COVID-PCD study)

|  | **nNO^a^** | **Biopsy** | **Genetic testing** |
| --- | --- | --- | --- |
|  | n = 671 | n = 724 | n = 724 |
|  | OR (95%CI) | OR (95%CI) | OR (95%CI) |
| **Year of diagnosis** |  |  |  |
| (reference category: < 2001) |  |  |  |
| 2001-2010 | 1.5 (0.94-2.3) | 1.95 (1.2-3.2) | 1.6 (1.1-2.6) |
| > 2010 | 2.2 (1.5-3.2) | 3.2 (2.1-4.9) | 4.7 (3.2-6.9) |
|  |  |  |  |
| **Situs abnormalities** |  |  |  |
| (reference category: no) |  |  |  |
| yes | 0.5 (0.4-0.7) | 0.5 (0.4-0.8) | 0.7 (0.5-0.94) |
|  |  |  |  |
| **Countries/regions** |  |  |  |
| (reference category: United Kingdom) |  |  |  |
| North America | 0.8 (0.5-1.3) | 0.2 (0.1-0.4) | 2.1 (1.3-3.5) |
| Germany | 1.8 (1.03-3.2) | 0.6 (0.3-1.3) | 1.9 (1.1-3.4) |
| Switzerland | 0.5 (0.2-1.1) | 0.4 (0.2-0.8) | 0.6 (0.3-1.2) |
| Italy | 1.005 (0.5-2.0) | 1.4 (0.5-4.1) | 1.3 (0.7-2.7) |
| France | 0.7 (0.3-1.5) | 0.2 (0.1-0.5) | 2.3 (1.1-5.5) |
| Australia | 0.7 (0.3-1.6) | 0.97 (0.3-3.6) | 0.7 (0.3-1.7) |
| Other European countries | 0.7 (0.4-1.4) | 0.4 (0.2-0.8) | 1.4 (0.8-2.4) |
| Other non-European countries | 0.4 (0.2-0.95) | 0.2 (0.1-0.5) | 0.6 (0.3-1.4) |

Abbreviations: nNO, nasal nitric oxide. Odds Ratio (OR) and 95% Confidence Interval (CI) reported. Odds Ratios were adjusted for all variables included in the multivariable model. Performed tests: Participants who report that the test was performed (“yes”) were compared to the group who reported either no test (“no”) or did not recall the test (“I don’t know” and missing). ^a^Only participants age >= 5 years are included.
