# Supplementary material for: Diagnostic testing in people with primary ciliary dyskinesia: An international participatory study
Source: PLOS Glob Public Health. 2023 Sep 11;3(9):e0001522. doi: 10.1371/journal.pgph.0001522 (PMC10495017; doi:10.1371/journal.pgph.0001522)
Supplement: S5 Table — Abbreviations: nNO, nasal nitric oxide. Adjusted Odds Ratio (OR) and 95% Confidence Interval (CI) reported. Odds Ratios were adjusted for all variables included in the multivariable model. aParticipants who report that the test was performed (“yes”) and who did not recall the test (“I don’t know” and missing) were compared to the group who reported no test (“no”). bOnly participants age > = 5 years are included. (DOCX) [file pgph.0001522.s005.docx]

**S5 Table.** Sensitivity analysis of factors associated with performed nNO, biopsy and genetic testing in which we assumed that everybody in the group “no recall” had the test done in the COVID-PCD study^a^

|  | **nNO^b^** | **Biopsy** | | **Genetic testing** | |
| --- | --- | --- | --- | --- | --- |
|  | n = 671 | | n = 724 | | n = 724 |
|  | OR (95%-CI) | | OR (95%-CI) | | OR (95%-CI) |
| **Year of diagnosis** |  | |  | |  |
| (reference category: < 2001) |  | |  | |  |
| 2001-2010 | 1.1 (0.7-1.8) | | 1.4 (0.8-2.5) | | 0.99 (0.6-1.5) |
| > 2010 | 1.2 (0.8-1.7) | | 1.6 (0.98-2.5) | | 2.8 (1.9-4.2) |
|  |  | |  | |  |
| **Situs abnormalities** |  | |  | |  |
| (reference category: no) |  | |  | |  |
| yes | 0.5 (0.4-0.7) | | 0.4 (0.3-0.6) | | 0.8 (0.5-1.1) |
|  |  | |  | |  |
| **Countries/regions** |  | |  | |  |
| (reference category: United Kingdom) |  | |  | |  |
| North America | 0.6 (0.4-1.1) | | 0.1 (0.1-0.3) | | 1.4 (0.8-2.3) |
| Germany | 1.5 (0.8-2.8) | | 0.3 (0.1-0.8) | | 1.2 (0.6-2.1) |
| Switzerland | 1.1 (0.5-2.4) | | 0.3 (0.1-1.1) | | 0.7 (0.3-1.4) |
| Italy | 0.6 (0.3-1.1) | | 0.5 (0.2-1.6) | | 1.1 (0.6-2.3) |
| France | 0.5 (0.2-1.1) | | 0.2 (0.1-0.5) | | 1.3 (0.6-3.2) |
| Australia | 0.5 (0.2-1.2) | | 0.5 (0.1-2.3) | | 0.6 (0.3-1.5) |
| Other European countries | 0.6 (0.4-1.1) | | 0.3 (0.1-0.6) | | 1.04 (0.6-1.8) |
| Other non-European countries | 0.4 (0.2-0.8) | | 0.1 (0.04-0.4) | | 0.4 (0.2-0.8) |

Abbreviations: nNO, nasal nitric oxide. Adjusted Odds Ratio (OR) and 95% Confidence Interval (CI) reported. Odds Ratios were adjusted for all variables included in the multivariable model. ^a^Participants who report that the test was performed (“yes”) and who did not recall the test (“I don’t know” and missing) were compared to the group who reported no test (“no”). ^b^Only participants age >= 5 years are included.
