# Supplementary material for: Diagnostic testing in people with primary ciliary dyskinesia: An international participatory study
Source: PLOS Glob Public Health. 2023 Sep 11;3(9):e0001522. doi: 10.1371/journal.pgph.0001522 (PMC10495017; doi:10.1371/journal.pgph.0001522)
Supplement: S6 Table — Abbreviations: nNO, nasal nitric oxide. Performed tests: Participants who report that the test was performed (“yes”) were compared to the group who reported either no test (“no”) or did not recall the test (“I don’t know” and missing). aOnly participants age > = 5 years are included. (DOCX) [file pgph.0001522.s006.docx]

**S6 Table.** Performance of nNO measurement, biopsy and genetic tests by year of diagnosis, situs abnormalities and countries, in people with primary ciliary dyskinesia (PCD) (COVID-PCD study)

|  | **nNO done^a^** | **No nNO done^a^** | **Brushing done** | **No brushing done** | **Genetics done** | **No genetics done** |
| --- | --- | --- | --- | --- | --- | --- |
|  | n = 342 | n = 351 | n = 561 | n = 186 | n = 435 | n = 312 |
|  | n (%) | n (%) | n (%) | n (%) | n (%) | n (%) |
| **Year of diagnosis** |  |  |  |  |  |  |
| < 2001 | 85 (37) | 146 (63) | 145 (63) | 86 (37) | 89 (39) | 142 (61) |
| 2001-2010 | 69 (47) | 77 (53) | 111 (76) | 35 (24) | 74 (51) | 72 (49) |
| > 2010 | 176 (60) | 118 (40) | 291 (84) | 56 (16) | 261 (75) | 86 (25) |
| Missing | 12 (58) | 10 (42) | 14 (60) | 9 (40) | 11 (58) | 12 (52) |
|  |  |  |  |  |  |  |
| **Situs abnormalities** |  |  |  |  |  |  |
| No | 219 (57) | 164 (43) | 326 (81) | 76 (19) | 262 (65) | 140 (35) |
| Yes | 123 (40) | 187 (60) | 235 (68) | 110 (32) | 173 (71) | 172 (29) |
|  |  |  |  |  |  |  |
| **Countries/regions** |  |  |  |  |  |  |
| United Kingdom | 76 (52) | 69 (48) | 128 (85) | 22 (15) | 76 (51) | 74 (49) |
| North America | 68 (48) | 74 (52) | 97 (61) | 61 (39) | 108 (68) | 50 (32) |
| Germany | 66 (66) | 34 (34) | 87 (81) | 20 (19) | 73 (68) | 34 (32) |
| Switzerland | 15 (35) | 28 (65) | 33 (69) | 15 (31) | 18 (38) | 30 (62) |
| Italy | 24 (49) | 25 (51) | 46 (87) | 7 (13) | 28 (53) | 25 (47) |
| France | 19 (46) | 22 (54) | 29 (66) | 15 (34) | 31 (70) | 13 (30) |
| Australia | 13 (46) | 15 (54) | 29 (88) | 4 (12) | 16 (48) | 17 (52) |
| other European countries | 47 (44) | 59 (56) | 87 (76) | 28 (24) | 68 (59) | 47 (41) |
| other countries | 14 (36) | 25 (64) | 25 (64) | 14 (36) | 17 (44) | 22 (56) |

Abbreviations: nNO, nasal nitric oxide. Performed tests: Participants who report that the test was performed (“yes”) were compared to the group who reported either no test (“no”) or did not recall the test (“I don’t know” and missing). ^a^Only participants age >= 5 years are included.
