# Supplementary material for: Diagnostic testing in people with primary ciliary dyskinesia: An international participatory study
Source: PLOS Glob Public Health. 2023 Sep 11;3(9):e0001522. doi: 10.1371/journal.pgph.0001522 (PMC10495017; doi:10.1371/journal.pgph.0001522)
Supplement: S7 Table — Abbreviations: nNO, nasal nitric oxide. Odds Ratio (OR) and 95% Confidence Interval (CI) reported. Performed tests: Participants who report that the test was performed (“yes”) were compared to the group who reported either no test (“no”) or did not recall the test (“I don’t know” and missing). aUnivariable analysis. bOnly participants age > = 5 years are included. (DOCX) [file pgph.0001522.s007.docx]

**S7 Table**. Factors associated with nNO measurement, biopsy and genetic tests, in people with primary ciliary dyskinesia (PCD), univariable (COVID-PCD study, N = 747)

|  | **nNO^a^** | **Biopsy** | **Genetic testing** |
| --- | --- | --- | --- |
|  | OR (95%CI) | OR (95%CI) | OR (95%CI) |
| **Age at diagnosis** | 1.01 (0.9995-1.02) | 1.00 (0.99-1.01) | 0.99 (0.98-0.99993) |
|  |  |  |  |
| **Year of diagnosis** |  |  |  |
| (reference category: < 2001) |  |  |  |
| 2001-2010 | 1.6 (1.02-2.4) | 1.9 (1.2-3.0) | 1.7 (1.1-2.5) |
| > 2010 | 2.6 (1.8-3.7) | 3.2 (2.1-4.7) | 4.9 (3.4-7.0) |
|  |  |  |  |
| **Situs abnormalities** |  |  |  |
| (reference category: no) |  |  |  |
| yes | 0.5 (0.4-0.7) | 0.5 (0.4-0.7) | 0.5 (0.4-0.7) |
|  |  |  |  |
| **Countries/regions** |  |  |  |
| (reference category: United Kingdom) |  |  |  |
| North America | 0.8 (0.5-1.3) | 0.3 (0.2-0.5) | 2.1 (1.3-3.4) |
| Germany | 1.8 (1.05-3.0) | 0.7 (0.4-1.5) | 2.1 (1.3-3.5) |
| Switzerland | 0.5 (0.2-0.97) | 0.4 (0.2-0.8) | 0.6 (0.3-1.1) |
| Italy | 0.9 (0.5-1.7) | 1.1 (0.5-3.0) | 1.1 (0.6-2.1) |
| France | 0.8 (0.4-1.6) | 0.3 (0.2-0.7) | 2.3 (1.5-4.9) |
| Australia | 0.8 (0.3-1.8) | 1.2 (0.4-4.5) | 0.9 (0.4-2.0) |
| Other European countries | 0.7 (0.4-1.2) | 0.5 (0.3-0.99) | 1.4 (0.9-2.3) |
| Other non-European countries | 0.5 (0.2-1.04) | 0.3 (0.1-0.7) | 0.8 (0.4-1.5) |

Abbreviations: nNO, nasal nitric oxide. Odds Ratio (OR) and 95% Confidence Interval (CI) reported. Performed tests: Participants who report that the test was performed (“yes”) were compared to the group who reported either no test (“no”) or did not recall the test (“I don’t know” and missing). ^a^Only participants age >= 5 years are included.
